# Occurrence and dietary risk assessment of chloramphenicol residues in honey products in Saudi Arabia

**DOI:** 10.1016/j.toxrep.2025.102066

**Published:** 2025-06-08

**Authors:** Lulu Almutairi, Abdullah AlSayari, Somaiah Almubayedh, Sarah AlSubaie, Thamer AlMudhehi, Malak Almutairi, Amani S. Alqahtani

**Affiliations:** a Executive Department of Research and Studies, Saudi Food and Drug Authority, Riyadh, Saudi Arabia; b Department of Reference Laboratory, Saudi Food and Drug Authority, Riyadh, Saudi Arabia

**Keywords:** Risk assessment, Regulation, Margin of exposure, LC-MS/MS, Antibiotic residues

## Abstract

**Background:**

Chloramphenicol (CAP) is a broad-spectrum antibiotic with potentially fatal side effects, including suspected carcinogenicity and toxicity in sensitive individuals. Due to these risks, CAP has been banned in food-producing animals, including honey, by regulatory authorities worldwide. This study investigates the presence of CAP residues in honey products from the Saudi market and evaluates the associated dietary exposure risk.

**Methods:**

A total of 902 honey samples collected during 2018–2019 were retrieved from the Saudi Food and Drug Authority (SFDA) to investigate honey safety and chemical components. CAP was extracted using a validated liquid-liquid extraction method and quantified using liquid chromatography-tandem mass spectrometry (LC-MS/MS). Dietary exposure was assessed using the margin of exposure (MOE) approach, with relevant data on honey consumption and body weight sourced from published studies.

**Results:**

CAP residues were detected in 54 (6 %) of the tested honey samples, with a mean concentration of 0.16 ± 0.032 µg/kg. The 95th percentile MOE for adults was 31,752 in the lower-bound scenario and 24,290 in the upper-bound scenario, exceeding the critical threshold of 10,000 established by the European Food Safety Authority, which indicates low public health concern and low priority for risk management.

**Conclusion:**

Our findings indicate a low health risk associated with honey consumption in Saudi Arabia, as MOE values exceeded the critical safety threshold. However, ongoing monitoring, stricter regulations, and enhanced awareness are recommended to ensure the safety and quality of honey products.

## Highlights

• CAP residues found in 6 % of Saudi honey samples.
• MOE for adults exceeded EFSA's safety threshold.
• Ongoing monitoring of honey safety is recommended.

## Graphical Abstract

**Figure:**
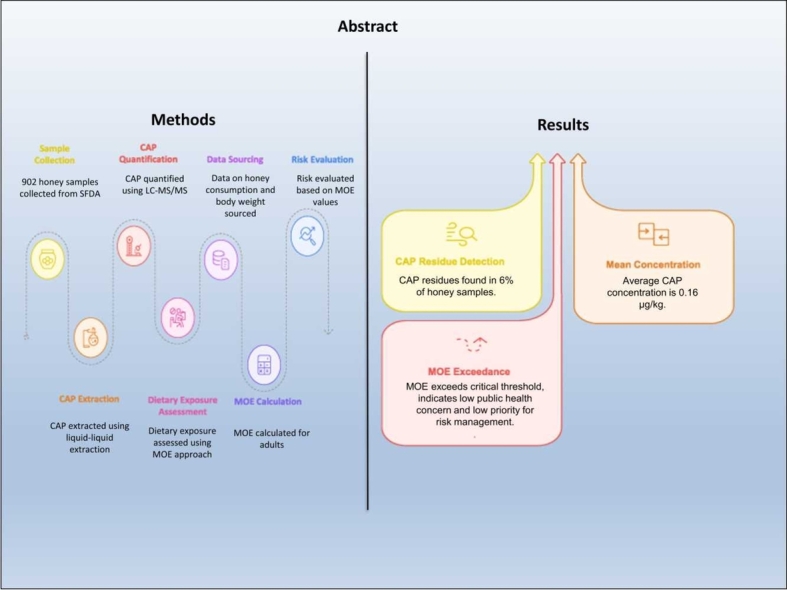

## 1 Introduction

Honey is a naturally sweet food that bees produce from sugary plant secretions (flower nectar) through regulated processes and particular enzymatic activity. Honey has been associated with various health benefits, such as boosting the immune system, aiding digestion, and promoting wound healing [1]. In Saudi Arabia, honey holds significant cultural and religious importance and is highly valued for its nutritional and medicinal properties. Notably, honey consumption in Saudi Arabia is high, with the average person consuming around 320 g annually, double the global average of 160 g per year [2], [3].

Although honey is well-known for its beneficial biological and therapeutic activities, it may contain contaminants, particularly antibiotics. In beekeeping, antibiotics are often used within hives to prevent or treat infectious diseases in honeybees [4]. However, this practice may result in antibiotic residues in bee products such as honey. The excessive use of antibiotics, including tetracyclines, sulfonamides, and Chloramphenicol (CAP), can further increase these residues, potentially posing health risks to consumers [4], [5]. Chloramphenicol, a broad-spectrum bacteriostatic antibiotic, is one such veterinary antibiotic occasionally detected in honey [6].The most serious adverse effects of CAP in humans include bacterial resistance, metabolic toxicity, and the risk of aplastic anemia and bone marrow suppression [7], [8], [9].

Given the potential impact of CAP on public health, the Codex Alimentarius Commission in 2002 stated that "because of the toxicity of CAP, a maximum residue limit cannot be established, and the substance should therefore not be used in food production [10]." Consequently, many countries, including Saudi Arabia, have adopted strict regulations to prevent CAP use in food-producing animals. The European Commission has also implemented reference points for action (RPA) for CAP in food. Initially set at 0.3 µg/kg, the RPA was later updated to 0.15 µg/kg in November 2022. These standards ensure that control laboratories can detect even trace amounts of CAP contamination in food products. It is worth mentioning that CAP may also occur naturally, as it is produced by the soil bacterium *Streptomyces venezuelae*. Plants can absorb CAP residues from the soil, further increasing the risk of consumer exposure [11]. Despite prohibiting chloramphenicol, several international studies that investigated food samples for CAP found positive samples [12], [13], [14]. Suggesting the needs of monitoring food products for prohibited antibiotic and residues.

In Saudi Arabia, the Saudi Food and Drug Authority (SFDA) regulates the honey industry to ensure consumer safety and product quality. Honey must comply with good manufacturing practices and be free of contaminants, such as pesticides, heavy metals, and bacteria. Furthermore, honey labeling must accurately reflect the product’s name, producer, country of origin, and production date. SFDA adheres to international food safety standards prohibiting CAP use by beekeepers.

To our knowledge, no research has been conducted to investigate CAP contamination in honey products in Saudi Arabia. This study aims to address this gap by determine the existence and concentration of chloramphenicol residues in honey products in Saudi Arabia, and measure the potential risk associated with consuming contaminated honey among the Saudi population.

## 2 Methods

### 2.1 Study design

A retrospective secondary data analysis was conducted to detect CAP residues in honey samples from Saudi Arabia, followed by a risk assessment using data collected in 2018 and 2019.

### 2.2 Sampling and sample analysis

#### 2.2.1 Sample collection

A total of 902 honey samples were collected during 2018 and 2019 from different sampling points, including local markets, manufacturers or port. The samples either were collected based on monitoring plans, suspicions or transferred from labs Samples were transported in suitable containers to SFDA laboratories in Riyadh, Jeddah, and Dammam. Upon arrival, each sample was examined to ensure it was sealed and intact, in accordance with sample selection requirements, before being stored at −20 °C until analysis. All samples were collected following CODEX general sampling guidelines [15].

#### 2.2.2 Sample extraction and clean-up (reagents and chemicals)

High-performance liquid chromatography (HPLC)-grade solvents were used for extraction and analysis. Methanol, ethyl acetate, and formic acid were obtained from Merck (Darmstadt, Germany). Chloramphenicol analytical standards and chloramphenicol-d5 internal standards were sourced from Cambridge Isotope Laboratories (Andover, MA, USA). Analyses were performed using the method described by the Austrian Agency for Health and Food Safety (2011) [16]. In a 15 mL polypropylene tube, 3 g of homogenized sample was weighed, followed by the addition of 5 mL of water. The mixture was incubated in a water bath at 40 °C for 10 min, then shaken for 10 min. Subsequently, 4 mL of water-saturated ethyl acetate was added and shaken for another 10 min. The sample was centrifuged at 4000 rpm for 5 min, and the supernatant was evaporated using a nitrogen evaporator system. The residue was dissolved in 1 mL of mobile phase, filtered through a 0.45 μm nylon syringe filter, and transferred to an analysis vial.

**Fig. 1:**
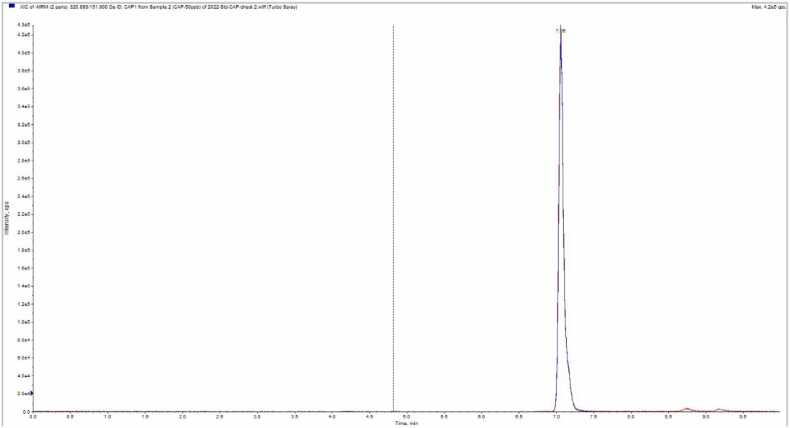
The chromatogram of separated chloramphenicol by LC-MS/MS.

#### 2.2.3 Chloramphenicol analysis using LC-MS/MS

Analyses were conducted using an Agilent 1290 Series liquid chromatography system equipped with an autosampler, binary pump, and column compartment, controlled by Analyst software. A Phenomenex Aqua C18 column (5 µm, 2.00 ×50 mm) was used to separate CAP at a flow rate of 0.4 mL/min. Gradient conditions employed methanol and 0.1 % formic acid as the mobile phase (Table 1). The column temperature was set to 40 °C, and the injection volume was 10 µL. Mass spectrometry analyses were performed using a 6500 Qtrap mass analyzer (AB Sciex, Canada), with nitrogen as the collision gas. The ion source operated at 550 °C with an electrode voltage of −4300 V. Multiple Reaction Monitoring (MRM) was used for MS detection in negative mode. The precursor ion for chloramphenicol was 321 *m/z*, with product ions at 257 *m/z* and 152 *m/z*. For chloramphenicol-d5, the precursor ion was 326 *m/z*, and the product ion was 157 *m/z* (Table 2).

**Table 1:** Mobile phase gradient timetable.

**Time (min.)**	**0.1 % formic acid in water**	**Methanol (%)**	**Flow rate (mL/min.)**
0	95	5	0.4
5	5	95	0.4
6	5	95	0.4
7	95	5	0.4
10	95	5	0.4

**Table 2:** MS/MS acquisition parameters for the chloramphenicol analysis on the AB SCIEX qtrap 6500 using ESI.

**Analyte**	**Precursor ion (*m/z*)**	**Product ion (*m/z*)**	**collision energy (v)**
Chloramphenicol	321	257	−23
	321	152	−17
Chloramphenicol-d5	326	157	−17

#### 2.2.4 Quality control

To ensure high accuracy and reliability, blank and spiked samples were prepared and analyzed with each batch. Chloramphenicol recovery in spiked samples ranged from 70 % to 120 %, with an expanded uncertainty of ≤ 20 %. Calibration curves had a minimum correlation coefficient (r2) of > 0.9995 across at least four levels. The limit of quantification (LOQ) was set as ten times the signal-to-noise ratio. The laboratories conducting the analyses were accredited under ISO 17025 for determining chloramphenicol in honey using LC-MS/MS.

### 2.3 Risk assessment for dietary exposure to chloramphenicol

The estimated daily intake (EDI) of chloramphenicol was calculated using the following equation [17]:

$$\mathrm{EDI}\left(µ\frac{\frac{g}{\mathrm{kg}}}{\mathrm{day}}\right)=\frac{\mathrm{Ci}\times \mathrm{IRi}}{\mathrm{BW}}$$

Where:

• Ci: Average concentration of chloramphenicol in honey samples (μg/kg). LOQ values were assigned to samples below the LOQ as recommended by the World Health Organization [18].
• IRi: Average daily honey consumption per capita (12.3 g/day for Saudi adults) [19].
• BW: Average body weight (70 kg) [20].
  The margin of exposure (MOE) approach was adopted to assess safety concerns using the following equation [21]:
  $$\mathrm{MOE}=\frac{\mathrm{RPA}}{\mathrm{EDI}}$$
  Where:
• RPA: Reference Point for Action for chloramphenicol (0.3 μg/kg body weight per day when samples were analyzed) [22].
• EDI: Estimated daily intake.

Lower and upper bound scenarios were evaluated, assigning LOQ values of zero and the LOQ itself, respectively, for samples below the detection limit. A MOE of ≥ 10,000 indicates low public health concern and low priority for risk management [23]. Monte Carlo simulations (10,000 iterations) were performed to account for uncertainty and variability, with health risk values reported at the 95th percentile [24].

### 2.4 Statistical analysis

Descriptive statistics for sample frequencies were calculated as numbers and percentages. Mean CAP concentrations in detected samples were expressed in μg/kg. Monte Carlo simulations and data treatments were performed using Microsoft Office Excel 2016.

## 3 Results

### 3.1 Descriptive data of the sample

Of the 902 collected honey samples, a significant proportion of the sample lacks specific country details (172 Sample). Saudi Arabia and Kyrgyzstan have the second and third highest sample included in the dataset, respectively. On the other hand, countries like Russia, Switzerland, Jordan, Tajikistan, Austria, Thailand, United Republic of Tanzania, Mexico and Myanmar have only one sample each. Further information is illustrated in Table 3.

**Table 3:** Honey sample origin country.

**Country**	**Count**
Not declared	172
Saudi Arabia	92
Kyrgyzstan	68
India	57
Spain	45
Turkey	37
Poland	36
New Zealand	33
Kuwait	30
Kazakstan	30
Pakistan	30
Australia	25
Germany	23
Italy	22
Romania	20
Slovenia	18
Netherlands	16
Egypt	13
Yemen	13
Bulgaria	12
United Arab Emirates	11
Hungary	10
New Caledonia	10
Tunisia	7
China	7
Hong Kong	7
Sudan	6
Slovakia	6
Ukraine	5
France	5
Bosnia And Herzegovina	5
Cyprus	4
United Kingdom	3
Lebanon	3
Aruba	2
Ethiopia	2
Croatia	2
Morocco	2
United States	2
Oman	2
Jordan	1
Tajikistan	1
Austria	1
Thailand	1
Tanzania	1
Mexico	1
Switzerland	1
Myanmar	1
Russian Federation	1
**Grand Total**	**902**

### 3.2 Samples detected with CAP

Out of 902 honey samples marketed in Saudi Arabia, 6 % (54 samples) tested positive for chloramphenicol residues. The detected concentrations of chloramphenicol in these positive samples varied widely, ranging from 0.05 to 193.00 µg/Kg. The mean concentration of chloramphenicol in the positive samples was 0.16 ± 0.032 µg/Kg, where 0.032 represents the expanded uncertainty associated with the measurement. For the purpose of this descriptive analysis, any sample with a concentration below the limit of quantification (LOQ) was assigned a value of zero.

Half of the honey samples detected with chloramphenicol were originally from Kyrgyzstan, 27 (50 %) and 7 (14 %) were from Saudi Arabia, more details of the origin of the honey samples detected with chloramphenicol are presented in Table 4.

**Table 4:** Country of origin of the honey samples detected with chloramphenicol (n = 54).

**Country**	**Frequency (N)**	**Percent**
Kyrgyzstan	27	50
Saudi Arabia	7	14
Unknown Origin	9	16.70
Kazakstan	4	7.40
Turkey	3	5.60
Egypt	1	1.90
Australia	1	1.90
Romania	1	1.90
Russian Federation	1	1.90
Total	**54**	**100**

**Table 5:** Risk assessment calculations of consuming honey contaminated with chloramphenicol.

			Estimate daily intake µg/Kg bw/day		MOE	
Sample Type	Scenario	Average results (µg/Kg)	Mean	P95	Mean	P95
Honey	Lower Bound	0.16	2.04 × 10^−8^	3.22 × 10^−8^	18438	31752
	Upper Bound**	0.21	2.7 × 10^−8^	4.23 × 10^−8^	13780	24290*

* Lower bound scenario, the value below LOQ assumed to be zero

** Upper bound scenario, the value below LOQ assumed to be the value of LOQ

### 3.3 Risk assessment for dietary exposure to chloramphenicol

The 95 % percentile of EDI of the CAP for adults in Saudi Arabia was estimated to be 3.22 × 10^−8^ µg/kg bw /day in the lower bound and the 95 % percentile of MOE in adults due to honey ingestion was calculated as 31,752. For the upper bound, the 95 % percentile of EDI of the CAP for adults in Saudi Arabia was estimated to be 4.23 × 10^−8^µg/kg bw /day and the 95 % percentile of MOE in adults due to honey ingestion was calculated as 24,290.

## 4 Discussion

This study analyzed data for the determination of CAP in honey samples from Saudi Arabia based on liquid chromatography coupled with tandem mass spectrometry. In addition, potential risk assessment associated with exposure to CAP detected in honey was measured. Results showed that out of 902 honey samples, 54 (6 %) were contaminated with CAP, with most of the contaminated samples originating from a single country, which is Kyrgyzstan. However, the findings indicated that there were no potential risks associated with the consumption of honey containing CAP.

Our findings indicate that a notable percentage of both local and imported honey samples marketed in Saudi Arabia CAP, despite its global prohibition for use in food-producing animals, including honeybees. This detection of CAP in honey is consistent with several international studies. For instance, similar to our results, research from Brazil [8] reported CAP detection in 13.9 % of honey samples, while a study from China [13] found CAP in 20.3 % of their samples. Furthermore, European surveillance data [25] has also consistently reported the presence of CAP residues in honey, often at lower prevalence rates depending on the region and year. Studies from Turkey [26] have similarly identified CAP in various blossom honeys, and research from Indonesia [27] found CAP in processed honey samples with levels ranging from 0.01 to 0.09 µg/kg. These widespread findings underscore the persistent challenge of CAP contamination in the global honey supply chain. In contrast to these findings, studies conducted in India [12], [28] analyzed a large number of honey products and reported no detectable traces of chloramphenicol residues in any of their samples. This variability between results suggests that the origin and specific practices within different regions can significantly influence contamination levels. This highlights the potential impact of stringent regulatory frameworks and effective enforcement, suggesting that strict, harmonized guidelines coupled with comprehensive education for beekeepers on the risks of CAP use can significantly improve compliance among companies and beekeepers.

Furthermore, our analysis revealed that nearly half (48 %) of the CAP-positive samples exceeded the recommended maximum RML of 0.3 µg/kg, a level often adopted internationally as a regulatory benchmark or RPA. This high proportion of samples exceeding established safety standards points to a clear violation and raises serious concerns about the adequacy of current control measures, both at the production and import levels. The detection of CAP in 7 (14 %) locally produced Saudi honey samples specifically emphasizes the pressing need for enhanced domestic surveillance and enforcement to ensure compliance with national regulations.

The dietary risk assessment showed that the Margin of Exposure (MOE) for Saudi adults was well above the safety threshold of 10,000 in both lower-bound and upper-bound scenarios (31,752 and 24,290, respectively). This MOE suggests a low concern for public health risks, consistent with EFSA guidelines [26]. However, it is important to note that the use of an RPA rather than a Benchmark Dose Lower Confidence Limit (BMDL10) introduces a level of uncertainty. EFSA has emphasized that an MOE of 10,000 or higher may not reliably indicate low risk in cases with significant uncertainties, particularly when insufficient toxicological data are available for more precise risk quantification. This limitation highlights the need for further research to establish health-based guidance values for CAP exposure

The findings also emphasize the critical role of veterinary pharmacies in controlling the availability of CAP, which remains accessible as a treatment for non-food-producing animals such as dogs, cats, and horses. Veterinary pharmacies play a crucial role in regulating the unjustified and excessive use of antibiotics in food-producing animals to minimize associated risks [29]. Consequently, it is essential to educate farmers about the dangers of using chloramphenicol in livestock to ensure public safety. An educational campaign should be initiated to raise awareness about the urgent public health risks related to the crossover use of specific drugs, including CAP. Notably, the Saudi Food and Drug Authority (SFDA) is actively working to promote awareness among honey producers, breeders, and marketers. They are emphasizing the importance of adhering to best practices in the marketing and trading of honey products. To support this initiative, the SFDA is collaborating with various ministries, including the Ministry of Environment, Water and Agriculture, and the Ministry of Municipal and Rural Affairs, to coordinate the production, marketing, and trading of both locally produced and imported honey in Saudi Arabia.

The findings underscore the need for continuous monitoring of CAP residues in honey to ensure food safety. Regulatory authorities should collaborate to establish and enforce stricter maximum residue limits, develop harmonized guidelines for international trade, and promote awareness among stakeholders in the honey production chain. Responsible use of veterinary drugs and adherence to best practices in apiculture are crucial for safeguarding public health and maintaining consumer trust in honey products.

This study has certain limitations. Honey consumption data and the average body weight of the Saudi population were sourced from a previously published study. Additionally, the data analyzed were collected in 2018–2019, and changes in honey production and importation practices may have occurred since then. These limitations may have introduced uncertainties in the dietary exposure assessment. Despite these limitations, this study represents the first comprehensive analysis of CAP residues in honey and their associated dietary risks in Saudi Arabia.

## 5 Conclusion

Our results showed that a number of samples were contaminated with CAP. The median CAP concertation in the detected samples was 0.28 µg/kg, and the 95 % percentile of MOE in adults due to honey ingestion was 24,290 in the upper bound scenario. The calculated MOE for adults in Saudi Arabia was found to be more than the critical limits. This study suggests the need to continuously monitor honey for CAP residues and develop the appropriate CAP regulations to guarantee consumers' maximum quality and safety.

## CRediT authorship contribution statement

**Abdullah AlSayari:** Writing – review & editing, Writing – original draft, Methodology, Formal analysis, Conceptualization. **Lulu Almutairi:** Writing – review & editing, Writing – original draft, Methodology, Formal analysis, Conceptualization. **Sarah AlSubaie:** Writing – review & editing, Formal analysis, Data curation. **Somaiah Almubayedh:** Writing – review & editing, Methodology, Formal analysis, Conceptualization. **Malak Almutairi:** Writing – review & editing, Writing – original draft, Formal analysis, Data curation. **Thamer AlMudhehi:** Writing – review & editing, Formal analysis, Data curation. **Amani S. Alqahtani:** Writing – review & editing, Supervision, Methodology, Formal analysis, Conceptualization.

## Funding

This research received no specific grant from any funding agency.

## Declaration of generative AI and AI-assisted technologies in the writing process

During the preparation of this work, the author used Grammarly AI editing service in order to enhance the language of the original manuscript. After using this tool/service, the author reviewed and edited the content as needed and takes full responsibility for the content of the published article.

## Declaration of Competing Interest

The authors declare that they have no known competing financial interests or personal relationships that could have appeared to influence the work reported in this paper.

## Data Availability

Data will be made available on request.
