# Supplementary material for: Occurrence and dietary risk assessment of chloramphenicol residues in honey products in Saudi Arabia
Source: Toxicol Rep. 2025 Jun 8;14:102066. doi: 10.1016/j.toxrep.2025.102066 (PMC12209939; doi:10.1016/j.toxrep.2025.102066)
Supplement: Supplementary file 1 — Supplementary material [file mmc1.docx]

**Supplementary file 1.** Reported value of chloramphenicol concentration in the detected samples of honey

| **Country** | **VALUE/MICROG_KG** |
| --- | --- |
| NOT DECLARED | 0.01* |
| KYRGYZSTAN | 0.02* |
| NOT DECLARED | 0.04* |
| KAZAKSTAN | 0.04* |
| SAUDI ARABIA | 0.05 |
| NOT DECLARED | 0.05 |
| KYRGYZSTAN | 0.06 |
| KAZAKSTAN | 0.08 |
| KYRGYZSTAN | 0.08 |
| KYRGYZSTAN | 0.09 |
| KYRGYZSTAN | 0.1 |
| SAUDI ARABIA | 0.14 |
| KAZAKSTAN | 0.14 |
| KYRGYZSTAN | 0.14 |
| KYRGYZSTAN | 0.14 |
| KYRGYZSTAN | 0.16 |
| NOT DECLARED | 0.2 |
| KYRGYZSTAN | 0.21 |
| KYRGYZSTAN | 0.22 |
| KYRGYZSTAN | 0.34 |
| SAUDI ARABIA | 0.405 |
| NOT DECLARED | 0.41 |
| KYRGYZSTAN | 0.5 |
| KYRGYZSTAN | 0.55 |
| KYRGYZSTAN | 0.82 |
| KYRGYZSTAN | 0.85 |
| EGYPT | 0.86 |
| KYRGYZSTAN | 1.06 |
| KYRGYZSTAN | 1.25 |
| NOT DECLARED | 1.64 |
| KYRGYZSTAN | 1.77 |
| KYRGYZSTAN | 1.93 |
| AUSTRALIA | 2.39 |
| KYRGYZSTAN | 4.67 |
| TURKEY | 4.77 |
| KYRGYZSTAN | 6.59 |
| SAUDI ARABIA | 16.14 |
| KYRGYZSTAN | 19.7 |
| KYRGYZSTAN | 23.7 |
| KAZAKSTAN | 23.79 |
| KYRGYZSTAN | 27.7 |
| NOT DECLARED | 0.04 |
| KYRGYZSTAN | 0.05 |
| ROMANIA | 0.0704 |
| TURKEY | 0.088 |
| TURKEY | 0.093 |
| KYRGYZSTAN | 0.163 |
| NOT DECLARED | 0.2 |
| Saudi Arabia | 0.7 |
| Saudi Arabia | 0.97 |
| Saudi Arabia | 1.14 |
| NOT DECLARED | 1.38 |
| KYRGYZSTAN | 5.27 |
| RUSSIAN FEDERATION | Missing |
| **Total** | **54** |

* Samples found to be less than the LOQ (<0.05) were not included in the average
